# Risk factors associated with death in infants <120 days old with severe pertussis: a case-control study

**DOI:** 10.1186/s12879-020-05535-0

**Published:** 2020-11-16

**Authors:** Cong Liu, Lin Yang, Yuwei Cheng, Hongmei Xu, Feng Xu

**Affiliations:** 1 Department of Infectious Diseases, Children’s Hospital of Chongqing Medical University, Chongqing, 400014 China; 2 National Clinical Research Center for Child Health and Disorders, Ministry of Education Key Laboratory of Child Development and Disorders, Chongqing, 400014 China; 3 Chongqing Key Laboratory of Pediatrics, Chongqing, 400014 China; 4 Department of Emergency, Children’s Hospital of Chongqing Medical University, Chongqing, 400014 China; 5 Department of Pediatric Intensive Care Unit, Children’s Hospital of Chongqing Medical University, Chongqing, 400014 China

**Keywords:** Infant pertussis, Leukocytosis, Pulmonary hypertension, Acellular diphtheria, tetanus, and pertussis vaccine (DTaP)

## Abstract

**Background and purpose:**

Pertussis is a serious infectious disease in young infants, and severe cases frequently cause death. Our study explored risk factors for death from severe pertussis.

**Method:**

A case-control study of infants with severe pertussis admitted to the paediatric intensive care unit (PICU) in the Children’s Hospital of Chongqing Medical University, China, from January 1, 2013, to June 30, 2019, was conducted. Pertussis was confirmed by clinical features and laboratory examinations. Severe pertussis was defined as patients with pertussis resulting in PICU admission or death. To understand the risk factors for death, we compared fatal and nonfatal cases of severe pertussis in infants aged < 120 days by collecting clinical and laboratory data.

**Results:**

The participants included 63 infants < 120 days of age with severe pertussis. Fifteen fatal cases were confirmed and compared with 44 nonfatal severe pertussis cases, Four patients with termination of treatment were excluded. In the univariate analysis, the risk factors associated with death included apnoea (*P* = 0.001), leukocytosis (white blood cell (WBC) count≥30 × 10^9^/L (*P* = 0.001) or ≥ 50 × 10^9^/L (*P* = 0)), highest lymphocyte count (*P* = 0), pulmonary hypertension (*P* = 0.001), and length of PICU stay (*P* = 0.003). The multivariate analysis revealed that apnoea (OR 23.722, 95%CI 2.796–201.26, *P* = 0.004), leukocytosis (OR 63.708, 95%CI 3.574–1135.674, *P* = 0.005) and pulmonary hypertension (OR 26.109, 95%CI 1.800–378.809, *P* = 0.017) were significantly associated with death.

**Conclusion:**

Leukocytosis and pulmonary hypertension exhibited the greatest associations with death in infants with severe pertussis admitted to the PICU. Vaccination is still the most effective protection method against pertussis.

## Introduction

**Fig. 1:**
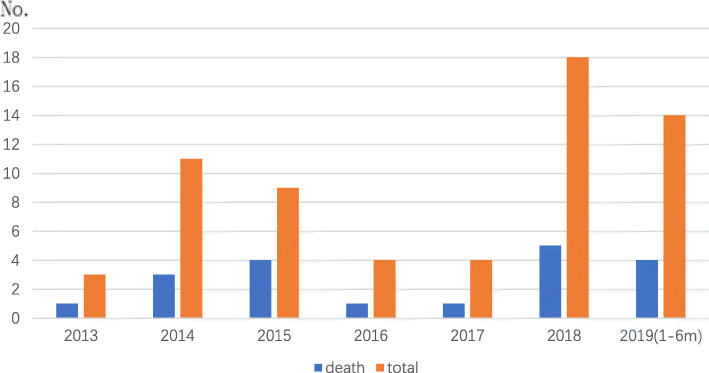
The number of pertussis cases needing PICU admission and number of deaths, Children’s Hospital of Chongqing Medical University, China; January 1, 2013- June 30, 2019

Pertussis is an acute respiratory infection caused by *Bordetella pertussis* and is characterized by paroxysmal coughing, coughing ending in a whooping sound, vomiting, apnoea or cyanosis [1]. The World Health Organization (WHO) estimated that 151,074 pertussis cases occurred in 2018 and 89,000 pertussis deaths occurred in 2008 (http://www.who.int/ith/diseases/pertussis/en/). However, even after widespread vaccination with the DTaP, reported pertussis cases substantially increased and are still increasing in China. The current programme for pertussis vaccination in China is administration of 1 dose at 3, 4, and 5 months of age and 1 increased dose at 18–24 months of age. At present, there is no programme for pertussis immunization during pregnancy [2, 3]. According to the Chinese Center for Disease Control and Prevention, 32,452 cases of pertussis were reported in 2011–2017, with an annual incidence 0.34 per 100,000 individuals; 60% of cases were in infants ≤5 months old. Hence, pertussis remains a serious disease in China, particularly in those too young to have completed their vaccination course. However, data on severe pertussis and death due to pertussis in China are scarce. Chongqing, a city in Western China, is among the top five cities with the highest pertussis prevalence [3]. To elucidate the status of severe pertussis in Chongqing, we collected data from January 2013 to July 2019 at the Children’s Hospital of Chongqing Medical University. The numbers of severe pertussis cases and deaths in < 120-day-old infants are shown in Fig. 1; both cases and deaths increased annually between 2016 and 2019. To gain further insight into the characteristics and treatment of severe pertussis and to determine the most important risk factors associated with death, we analysed data from < 120-day-old infants with severe pertussis.

## Methods

### Cases and controls

This was a case-control study of paediatric pertussis patients admitted to the PICU at the Children’s Hospital of Chongqing Medical University, China, from January 1, 2013 to June 30, 2019. The clinical features of pertussis included increasing frequency and severity of cough ending in a whooping sound or vomiting, apnoea or cyanosis. Some newborns had only cyanosis without cough. All cases were confirmed by isolation of *B. pertussis* by culture or detection of *B. pertussis* DNA by polymerase chain reaction (PCR) assays. Severe pertussis was defined as patients with pertussis resulting in PICU admission. The case group was defined as pertussis patients admitted to the PICU and dying from pertussis disease. The control group included pertussis patients admitted to the PICU and surviving pertussis disease.

### Medical record review

Hospital medical records were obtained, and data on birth factors; vaccination history; symptoms; course of illness; comorbidities; treatment including exchange transfusion (ET) or extracorporeal membrane oxygenation (ECMO); type of respiratory support; patient outcome; microbiology test results, such as full blood count; chest imaging reports by radiologists; and echocardiography findings were recorded. The birth weight and gestational age were obtained from birth records. Vaccination status was collected from the medical record or a copy of the vaccination record. Fever was defined as a tympanic or axillary temperature above 38 °C. The course of illness included the cough duration before hospitalization, PICU admission and the length of hospital and PICU stay, which were collected from the medical records. Pneumonia was defined by chest imaging. The highest white blood cell (WBC) count, platelet count, and lymphocyte count were recorded and analysed. Leucocytosis was defined as a WBC count ≥30 × 10^9^/L.

### Statistical analysis

SPSS v 23.0 software was used for the statistical analysis. The basic information and clinical characteristics of the two groups were compared by chi-square tests for categorical variables and Student’s t-tests or Mann Whitney U tests for continuous variables. For normally distributed continuous variables, Student’s t-test was used for comparisons; otherwise, a Mann Whitney U test was used. Univariate logistic regression analysis was performed to identify factors associated with death among infants with pertussis disease requiring admission to the PICU. Backward elimination stepwise multivariable logistic regression models were developed to identify independent risk factors for death among infants with pertussis disease requiring admission to the PICU. Th P-to-remove value was set as a *c* > 0.05. Variables with a *P* value of < 0.05 were included in the final regression model. All *P* values < 0.05 were considered statistically significant for all tests.

## Results

From January 1, 2013 to June 30, 2019, 3280 pertussis patients were admitted to the Children’s Hospital of Chongqing Medical University, and 89 patients with severe pertussis were admitted to the PICU, including 63 patients who were < 120 days old. The 63 patients included 1 patient aged ≤1 month, 31 patients (49.2%) aged 1–2 months, 24 patients (38%) aged 2–3 months, and 7 patients (11.1%) aged 3–4 months, and the average age of cough onset was < 3 months. For 5 patients, treatment was terminated by their parents because the condition too serious to survive, and their parents were unable to accept the bad consequences. Among those who stopped receiving treatment, 4 patients died and 1 patient survived according to telephone follow-up. The 4 patients who died were excluded, and 59 cases were included in our study. The basic information, clinical symptoms and vaccination history of the patients are presented in Table 1.

**Table 1:** Basic information, clinical symptoms and vaccination history of the death group and nondeath group

Characteristic	Deaths(*n* = 15)		Nondeaths(*n* = 44)		
	NO. of Patients	NO.(%)	NO. of Patients	NO.(%)	*P* Value
age					
≤ 1 month	1	6.67%	1	2.27%	0.417
1-2 month	6	40%	19	43.18%	0.829
2-3 month	4	26.67%	13	29.55%	0.832
3-4 month	4	26.67%	11	25.00%	0.898
Male sex	6		26		0.2
weight (kg)		5.13 ± 1.12		5.27 ± 1.07	0.135
premature	4	26.67%	6	13.64%	0.245
Birth weight (kg)		2.96 ± 4.41		3.17 ± 5.07	0.149
Vaccination history	2 (1 dose)		0		
symptoms					
fever	9	60.00%	19	43.18%	0.26
paroxysmal coughing	14	93.33%	43	97.70%	0.417
whoop	11	73.30%	23	52.30%	0.154
apnoea	12	80.00%	14	31.80%	0.001
cyanosis	15	100%	44	100%	
Laboratory confirmed	15	100%	44	100%	

Prematurity was defined as birth at < 37 weeks’ gestation

Only 2 infants had received 1 dose of the vaccine (DTaP) in the death group

Fever was defined as a tympanic or axillary temperature >38 °C

Fifteen of the 59 patients died (25.4%). We divided the patients into two groups: the fatal group (*n* = 15) and nonfatal group (*n* = 44). Thirty-two (54.2%) infants were male, including 6 in the fatal group and 26 in the nonfatal group. The average weight was 5.13 ± 1.12 kg in the fatal group and 5.27 ± 1.07 kg in the nonfatal group. There were no differences in prematurity,birth weight and month age between two groups. The vaccination acceptance rate was generally low in our study, as the onset age for all the patients in our study was less than 3 months; the vaccination rate was 3.3%, and only 2 infants had received 1 dose of the vaccine in the fatal group. Almost all (57/59) infants had cough and cyanosis. Only 2 patients had only cyanosis without coughing which one was newborn.

The clinical manifestations of pertussis, main laboratory indexes, complications and treatments are presented in Table 2. The majority of infants (*n* = 49) received echocardiograms, and congenital heart disease (CHD) (*n* = 20) and pulmonary hypertension (*n* = 11) were present in some infants. Although some patients had CHD, such as an atrial septal defect (ASD, *n* = 18), or patent ductus arteriosus (PDA, *n* = 2) without complex CHD or cardiac dysfunction, the rate of CHD was not significantly different between the fatal and nonfatal groups. Five patients in the fatal group and 3 patients in the nonfatal group had CHD and pulmonary hypertension. All 8 patients had an ASD. In addition to CHD, 16 patients had other comorbidities including bronchopulmonary dysplasia (*n* = 4), hypoxic ischaemic encephalopathy (*n* = 4), rotavirus enteritis (*n* = 4), Down syndrome (*n* = 1), hypoplasia of the laryngeal cartilage, adenovirus encephalitis, and epilepsy. Although the majority of patients had more than one comorbidity, none of the comorbidities were the main cause of death in our study.

**Table 2:** Course of illness, main laboratory indexes and treatment of the death group and nondeath group

Characteristic	Deaths (*n* = 15)		Nondeaths (*n* = 44)		
	NO. of Patients	NO. (%)	NO. of Patients	NO. (%)	*P* Value
Course of illness					
length of hospital stay (IQR)		10 (3–15)		25.5 (19.25–31.75)	0
Cough duration before the PICU (IQR)		11 (7–14)		14 (8–19.75)	0.061
length of PICU stay (IQR)		3 (2–12)		13.5 (8–17)	0.003
Leukocytosis (WBC ≥ 30 × 10^9^ /L.)	14	93.33%	19	43.18%	0.001
Leukocytosis (WBC ≥ 50 × 10^9^ /L.)	13	86.67%	7	15.91%	0
Highest WBC count		77.33 ± 28.56		30.52 ± 16.46	0
Highest lymphocyte count		29.59 ± 13.59		16.19 ± 9.20	0
Highest Platelet count		740.47 ± 199.93		696.05 ± 153.47	0.375
Congenital heart disease (ASD or PDA)	5 (13)	38.46%	15 (36)	41.66%	1
Pulmonary hypertension	8 (14)	57.14%	3 (36)	8.33%	0.001
Pulmonary hypertension without CHD	3 (8)	44.40%	0 (3)	0	0.214
Pneumonia	15	100%	44	100%	
Co-infectious with clearly pathogen	13	86.60%	38	86.36%	0.976
Consolidation of lung	9	60.00%	23	52.27%	0.604
Convulsions	2	13.30%	8	18.20%	0.666
Pertussis encephalopathy	5	33.30%	21	47.70%	0.332
Co- morbidity	12	80.00%	24	54.54%	0.081
Treatment					
Received macrolide antibiotics	15	100%	44	100%	
Days to macrolide initiation (IQR)		8 (6–11)		10.5 (9–15)	0.012
Received steroids	6	40.00%	13	29.50%	0.454
Respiratory support (invasive or/and noninvasive)	15	100%	36 + 4	90.90%	0.226
intubated	15	100%	36	81.80%	0.076
Ventilator time		3 (2–12)		8 (5–13.75)	0.412
Exchange transfusion	3	20.00%	2	4.55%	0.063
ECMO	1	0.07%	0		0.084

*Abbreviations*: *IQR* interquartile range, *CHD* congenital heart disease, *ECMO* extracorporeal membrane oxygenation, *ASD* atrial septal defect, *PDA* patent ductus arteriosus

Leukocytosis was defined as WBC count ≥30 × 10^9^ /L

Co-morbidity included CHD, bronchopulmonary dysplasia, hypoxic ischaemic encephalopathy, rotavirus enteritis, Down syndrome, hypoplasia of the laryngeal cartilage, adenovirus encephalitis, and epilepsy

All patients underwent chest imaging, and pneumonia and respiratory pathogens were detected by culture and PCR assays performed on sputum; serological examination was performed supplementally. Fifty-one patients (86%) had coinfections with clear pathogeny on admission; coinfections included respiratory syncytial virus (RSV) (*n* = 13), *Klebsiella pneumoniae* (*n* = 13), *Haemophilus influenzae* (*n* = 13), adenovirus (*n* = 3), and influenza B (*n* = 3). The majority of patients had more than one pathogen infection. However, the rates of coinfection with specific pathogens were not significantly different between the fatal and nonfatal groups.

During treatment, all the patients received macrolides. In the nonfatal group, 40 patients (90%) required respiratory support (36 were intubated and 4 required noninvasive ventilation). All fatal cases required intubation. Five patients were treated with ETs, and 3 died. The peak WBC count in patients in the fatal group with ET ranged from 70.44 × 10^9^/L to 84.93 × 10^9^/L. The peak WBC count in patients in the nonfatal group with ET ranged from 56.44 × 10^9^/L to 59.95 × 10^9^/L. All patients in the fatal group with ET experienced shock and respiratory failure, and 1 infant had multiorgan failure prior to the ET procedure. Two infants underwent ET twice to decrease the leukocyte count but subsequently died.

Only 1 patient in the fatal group received ECMO for 2 days and received respiratory support and haemodialysis (for renal insufficiency) at the same time. The infant’s WBC count was 97.22 × 10^9^/L on admission and peaked at 135.58 × 10^9^/L. The case was complicated with pulmonary hypertension, acute respiratory distress syndrome (ARDS), and multiorgan failure prior to the ECMO procedure. Finally, pulmonary haemorrhage and brain hernia occurred, and the patient died soon after.

The univariate analysis of risk factors related to death is shown in Table 3. According to the univariate analysis, the risk factors associated with death were apnoea (*P* = 0.001), highest lymphocytosis count (*P* = 0), WBC counts ≥30 × 10^9^/L (*P* = 0.001) and ≥ 50 × 10^9^/L(*P* = 0), pulmonary hypertension (*P* = 0.001), and length of PICU stay (days) (*P* = 0.003). The multivariate analysis of risk factors related to death is shown in Table 4. Apnoea (OR 23.722, 95%CI 2.796–201.26, *P* = 0.004), leukocytosis (OR 63.708, 95%CI 3.574–1135.674, *P* = 0.005) and pulmonary hypertension (OR 26.109, 95%CI 1.800–378.809, *P* = 0.017) were significantly associated with death. WBC counts ≥30 × 10^9^/L or ≥ 50 × 10^9^/L and pulmonary hypertension were significantly associated with death.

**Table 3:** Univariate logistic regression analysis of severe pertussis associated with death

characteristic	*P* Value	unadjusted OR(95%CI)
apnoea	0.001	8.571 (2.082–35.294)
length of PICU stay (IQR)	0.003	
Leukocytosis (WBC ≥ 30 × 10^9^ /L.)	0.001	18.421 (2.223–152.647)
Leukocytosis (WBC ≥ 50 × 10^9^ /L.)	0	34.357 (6.316–186.900)
Highest WBC count	0	
Highest lymphocyte count	0	
Pulmonary hypertension	0.001	14.667 (3.001–71.678)

*Abbreviations*: *IQR* interquartile range, *OR* odds ratio;CI, confidence interval

Leukocytosis was defined as WBC count ≥30 × 10^9^ /L

**Table 4:** Multivariate logistic regression analysis of severe pertussis associated with death

	adjusted OR	95%CI	*P* Value
apnea	23.722	2.796–201.260	0.004
Leukocytosis	63.708	3.574–1135.674	0.005
Pulmonary hypertension	26.109	1.800–378.809	0.017

*Abbreviations*: *CI* confidence interval, *OR* odds ratio

Leukocytosis was defined as WBC count ≥30 × 10^9^ /L

## Discussion

Pertussis resurgence has been observed in recent years, and severe pertussis and death due to pertussis have gradually increased. According to other research, age ≤ 3 months is a risk factor for severe and death in pertussis cases [4–9], and 78.8% (63/89) of severe pertussis patients were aged ≤3 months in our hospital; this result is consistent with other studies. Our findings were consistent in 59 patients with severe pertussis who had a cough onset age of < 3 months after excluding 4 patients for whom treatment was terminated and who later died. Our study analysed 15 fatal pertussis cases and 44 nonfatal pertussis cases. In the study by Winter et al., patients with fatal outcomes had a younger gestational age and lower birth weight than those with nonfatal outcomes [10]. The study by Abu-Raya et al. found age of < 4 weeks, prematurity, and female sex were independent risk factors for death [11].

We found that apnoea, leukocytosis, and pulmonary hypertension were significantly associated with death in severe pertussis cases. Currently, in severe pertussis cases, leukocytosis and lymphocytosis are commonly observed and are significantly correlated with death [6, 10, 12–15]. Pertussis toxin (PT) indirectly leads to the development of pulmonary hypertension through the induction of lymphocytosis (leukocytosis). The aggregation of leukocytes in pulmonary arterioles, veins and the lymphatic system was observed in the lungs of pertussis-infected infants after death [16–18]. In our study, leukocytosis was significantly associated with death, similar to the results of other publications. Only 2 patients with fatal outcomes did not present leukocytosis in our study. One patient had comorbid trisomy 21 syndrome with an ASD and a maximum WBC count of 16.58 × 10^9^/L. This infant was coinfected with RSV and diagnosed with severe pneumonia and ARDS. The second infant was admitted after cardiopulmonary resuscitation due to asphyxia. Pertussis was diagnosed after admission. The maximum WBC count was 23.19 × 10^9^/L. It is possible that asphyxia and leukocytosis were significantly related to pertussis infection. Both patients died of respiratory failure without pulmonary hypertension.

Of greatest importance in the treatment of pertussis is the administration of a macrolide antibiotic. PICU management of infant pertussis cases has been limited [17, 19, 20]. Extreme leukocytosis with lymphocytosis is associated with infant death [10]. Pertussis toxin (PT)-promoted leukocytosis and indirect development of pulmonary hypertension are the most important risk factors related to death in patients with severe pertussis. Could death be reduced by rapidly reducing leukocytes and lymphocytes in patients with severe pertussis? In 2004, Romano et al. published the first report on ET in a patient with severe pertussis [21]; thereafter, ET has been reported in multiple case reports of severe pertussis treatment [22–28]. According to previous research, although there is a lack of a unified standard, ET showed potential therapeutic benefits and promising results and is worthy of future exploration [18, 21, 25, 27, 29]. In our study, 5 patients were treated with ET, of whom 3 died. The peak WBC count of patients in the fatal group who underwent ET was significantly higher than that in the nonfatal group. Although there was no significant difference between the two groups treated with ET, the variations may be related to the time of treatment onset and the severity of illness; however, this needs to be confirmed by additional standardized control studies in the future. ECMO has a long history of use in the treatment of severe pertussis in young infants, but there is no consensus on its efficacy; however, it has been shown to have some clinical benefits [26, 27, 30–32]. In our research, ECMO was performed in only 1 patient in the fatal group. We observed no significant difference between the two groups treated with ECMO and ET, likely because these treatments were initiated only when the disease became life-threatening; however, these treatments still might play a potential role in severe pertussis.

To date, the main preventive strategy for pertussis in China is a vaccination scheme with three doses starting at 3 months of age. According to previous reports, most patients with fatal outcomes were too young to receive their first dose of the DTaP vaccine [10]. In our study, the majority of our infants were < 3 months of age, but 2 patients received one dose of the pertussis vaccine. Although the two patients were vaccinated, they began to cough before vaccination, and pertussis infection was considered. For this reason, many countries have already recommended that the initial vaccination scheme should start at 6 weeks of age. Since 2011, many countries have vaccinated pregnant women between 27 and 36 weeks gestation with the DTaP vaccine to prevent infection and death in infants ≤2 months old [33–38]. The DTaP vaccine has shown no adverse effects on the pregnancy or the foetus [39–41]. Therefore, our national vaccination schedule may need to be changed to protect an increased number of infants.

Because this is a retrospective study, it has some limitations, including a limited number of cases. All the patients in our study had varying degrees of hypoxemia and required respiratory support (59 invasive and 4 noninvasive); thus, their conditions were very serious, and a high mortality rate was observed, which explains why we had a limited number of cases. In addition, 10 infants did not undergo echocardiography, and heart and pulmonary artery conditions were unknown. Meanwhile, we could not collect the smoking habits of patients’ parents to determine whether any connection existed between pertussis and smoking.

## Conclusion

Throughout history, most pertussis deaths have occurred in infants. Leukocytosis and pulmonary hypertension are the greatest risk factors for death in infants with severe pertussis. Currently, the treatment of severe pertussis is limited and should be improved. ET and ECMO are also worth exploring as treatments, but additional data are needed. Vaccination is still the most effective protection method. Considering that ≤3 months of age is a risk factor for severe pertussis, our national vaccination schedule needs to be changed to protect infants in this age group.

## Data Availability

The datasets generated and/or analyzed during the current study are not publicly available due to participant privacy but are available from the corresponding author on reasonable request.

## Abbreviations

PICU: Paediatric intensive care unit
DTaP: Acellular diphtheria, tetanus, and pertussis vaccine
WHO: World Health Organization
PCR: Polymerase chain reaction
ET: Exchange transfusion
ECMO: Extracorporeal membrane oxygenation
WBC: White blood cell
CHD: Congenital heart disease
ASD: Atrial septal defect
PDA: Patent ductus arteriosus
IQR: Interquartile range
OR: Odds ratio
CI: Confidence interval
RSV: Respiratory syncytial virus
ARDS: Acute respiratory distress syndrome
PT: Pertussis toxin
